# Influence of Preparation pH for Superior Soot Oxidation: A Kinetic Perspective of K‑OMS‑2

**DOI:** 10.1021/acsomega.5c03869

**Published:** 2025-10-22

**Authors:** Nithya Rajagopal, Vikram Ashok Lokhande, Harshini Dasari, Nethaji Sundarabal

**Affiliations:** Department of Chemical Engineering, Manipal Institute of Technology, 125853 Manipal Academy of Higher Education, Manipal 576104, India

## Abstract

K-OMS-2, a tunnel-structured manganese oxide, has gained significant attention as a catalyst for soot oxidation due to its high redox capability and oxygen mobility. This study investigates the influence of the synthesis pH on the physicochemical properties of cryptomelane and its catalytic activity in soot oxidation. Two samples, synthesized at pH 3 and pH 5, were characterized using XRD, SEM, TEM, H_2_-TPR, and XPS. The pH5 sample exhibited higher crystallinity, an increased Mn^3+^/Mn^4+^ ratio of 0.41, and a greater O_ads_/O_latt_ ratio of 0.86, indicating enhanced redox behavior and oxygen mobility. TGA-based soot oxidation tests showed that the pH5 catalyst achieved a *T*_50%_ of 368 °C, compared to 389 °C for pH3 and 592 °C for uncatalyzed soot, indicating superior low-temperature activity. Kinetic analysis using Flynn–Wall–Ozawa (FWO) and Coats–Redfern (CR) models revealed a lower apparent activation energy for the pH5 sample (*E*_a_ = ∼130 kJ/mol) compared with the pH3 sample (*E*_a_ = ∼150 kJ/mol). These results confirm that synthesis pH significantly influences the structural and redox properties of cryptomelane and that catalysts with higher surface oxygen species and improved oxygen vacancy density exhibit enhanced catalytic performance.

## Graphical abstract

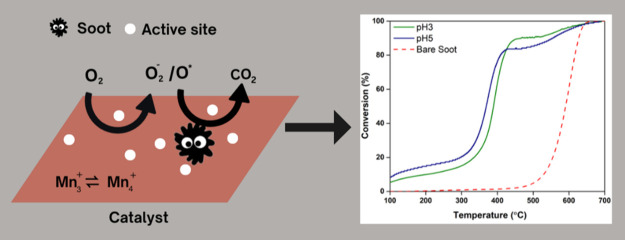

## 1 Introduction

In recent years, octahedral molecular sieves (OMS) have emerged as a promising alternative to traditional catalysts. OMS are microcrystalline materials characterized by a distinctive tunnel-like structure that offers unique catalytic properties.^1–4^ These materials are advantageous due to their ability to enhance catalytic efficiency and selectivity, making them a viable option for reducing soot emissions.

Among the various OMS, KOMS-2, or potassium-doped OMS, stand out for their exceptional catalytic properties. Kumar et al.^5^ employed acid-exchanged K-OMS-2 for the liquid-phase oxidation of cyclohexane with *t*-butyl hydroperoxide as the oxidant. The catalyst achieved high conversion rates (up to 97%) and selectivity (over 83%) for cyclohexanol and cyclohexanone. Furthermore, K-OMS-2 catalysts have shown excellent activity in the catalytic oxidation of toluene. The presence of lattice oxygen as both adsorption and active catalytic sites plays a crucial role in this process.^6,7^ K-OMS-2, with its unique structure and chemical composition, exhibits excellent performance in oxidation reactions.^8^ Iyer et al.^9^ synthesized K-OMS-2 along with doped K-OMS-2 to investigate propanol oxidation. The catalysts showed conversions ranging from 5% to 50%, with 100% selectivity to acetone. Therefore, K-OMS-2 is a versatile catalyst with specific advantages that include enhanced catalytic activity due to the enhanced redox property and durability. These parameters are crucial for a catalyst for its efficient soot oxidation activity at a lower temperature.^10–12^

Catalysts prepared under different pH conditions exhibit varied structural properties, which in turn affect their catalytic activity. For instance, MnO_*x*_-CeO_2_ oxides prepared under mildly acidic conditions (pH = 4) showed a significant decrease in the oxidation temperature, enhancing the soot oxidation efficiency by more than 150 °C compared to uncatalyzed soot oxidation.^13^ This improvement is attributed to better oxygen vacancy formation and enhanced oxygen exchange between the gas phase and the lattice oxygen species in the catalyst. The pH during catalyst preparation can also affect the surface properties, such as the surface area and the dispersion of active sites. For instance, ceria-based catalysts synthesized at different pH levels resulted in various shapes and structural properties, which influenced their soot oxidation efficiency.^13,14^ Catalysts with better soot-catalyst contact conditions, often achieved through optimal pH conditions, exhibited a higher catalytic activity. Catalysts prepared under specific pH conditions can also exhibit improved thermal stability and regeneration capabilities. Ru/Al_2_O_3_ catalysts demonstrated superior oxygen activation and regeneration abilities, which are crucial for maintaining high catalytic activity over multiple cycles.^15^ The pH of the catalyst plays a crucial role in determining the structural, surface, and catalytic properties of the catalysts, thereby significantly affecting their performance in soot oxidation reactions.

This study focuses on the effect of the pH of K-OMS-2 on the soot oxidation activity. This study focuses on investigating the kinetics of catalytic soot oxidation using different catalysts and evaluating their effectiveness through activation energy and pre-exponential factor calculations.

## 2 Experimental Procedures

### 2.1 Synthesis Method

Potassium permanganate and manganese sulfate were added to two separate beakers containing distilled water. The solutions were mixed, and concentrated HNO_3_ was added. The mixture was transferred to a Teflon-lined autoclave and heated at 100 °C for 24 h. The precipitate was washed with distilled water, filtered, and then dried at 120 °C. The dried sample was calcined at 500 °C for 4 h. During the washing of the precipitate, the pH was adjusted to 3 and 5, and the samples were named pH3 and pH5, respectively.

### 2.2 Characterization Techniques

XRD analysis was carried out in a Rigaku instrument using Cu-Kα radiation (λ = 1.54 Å). The XRD spectra of the as-synthesized samples were recorded in the 2θ range 20–90° at a scanning rate of 2°/min. Field emission scanning electron microscopy (FESEM) was performed using a Gemini 300, Carl Zeiss (Germany), equipped with a Schottky-type field emitter. The instrument provides a resolution of 0.7 nm at 15 kV and 1.2 nm at 1 kV. The measurements were carried out at the Central Instrumentation Facility of NITK, Surathkal. Raman spectra were recorded (Airix Corp, STR 500, Japan) in the wavenumber range of 200–4000 cm^–1^ using a laser as an exciting source of wavelength 532 nm with a resolution of <0.5 cm^–1^. H_2_ TPR analysis, acidity, and basicity analysis were carried out in a high-pressure chemisorption unit, Autochem 2950 (Micromeritics), equipped with a thermal conductivity detector. The soot-temperature-programmed reduction (TPR) procedure was carried out under a nitrogen atmosphere, employing a gas flow rate of 60 mL/min to understand the reducibility of the catalyst. The mixed sample was subjected to heating within a temperature span from 50 to 800 °C. This method facilitated the identification of active oxygen species present on the catalyst surface. X-ray photoelectron spectroscopy (XPS) was performed by using a SPECS instrument (Germany) with Al Kα radiation (1486.6 eV) as the excitation source. The binding energies were calibrated relative to C 1s at 284.6 eV. Peak fitting and deconvolution of the spectra were carried out using XPSPEAK41 software. The measurements were performed at PR Testing Services Lab.

### 2.3 Catalytic Activity and Kinetic Analysis

The thermogravimetric analyzer analyzed the catalytic soot oxidation of the synthesized samples. The catalyst and soot were combined in a ratio of 10:1 (mass ratio), and around 15 mg of the mixed sample was fed into the instrument. The analysis was carried out utilizing a thermogravimetric analyzer (specifically, a TA 55) with a zero air flow of 60 mL/min and a heating rate of 10 °C/min, ranging from room temperature to 700 °C.

To determine the kinetic triplets—pre-exponential factor (*A*), activation energy (*E*_a_), and reaction model—a solid-state reaction kinetics analysis is carried out. The TGA was used to measure the soot oxidation kinetics at various heating rates: 5, 10, 15, and 20 °C/min. The KAS approach and the Flynn–Wall–Ozawa (FWO) method were utilized to calculate activation energy (*E*_a_). The Coats–Redfern (CR) approach is one of the most often used nonisothermal models fitting techniques for Arrhenius factor and activation energy determination. The pre-exponential factor was determined using the Avrami method. The optimal reaction model for solid–gas reactions is identified by comparing computed and experimental values on a master plot.

## 3 Results and Discussion

### 3.1 Structural and Morphological Analysis

The XRD patterns in Figure 1 indicate that all of the synthesized samples exhibit a crystalline structure. The prepared samples exhibited a tetragonal phase of K-OMS-2 (00-042-1348). The diffraction peaks of 2θ = 14.53°, 20.72°, 33.04°, 43.66°, 48.54°, 52.71°, 57.98° correspond to standard data of K-OMS-2.^16^ There was no other extra peak seen, indicating the purity of the samples. The average crystallite size, volume, and lattice parameters are presented in Table 1.

**Figure 1:**
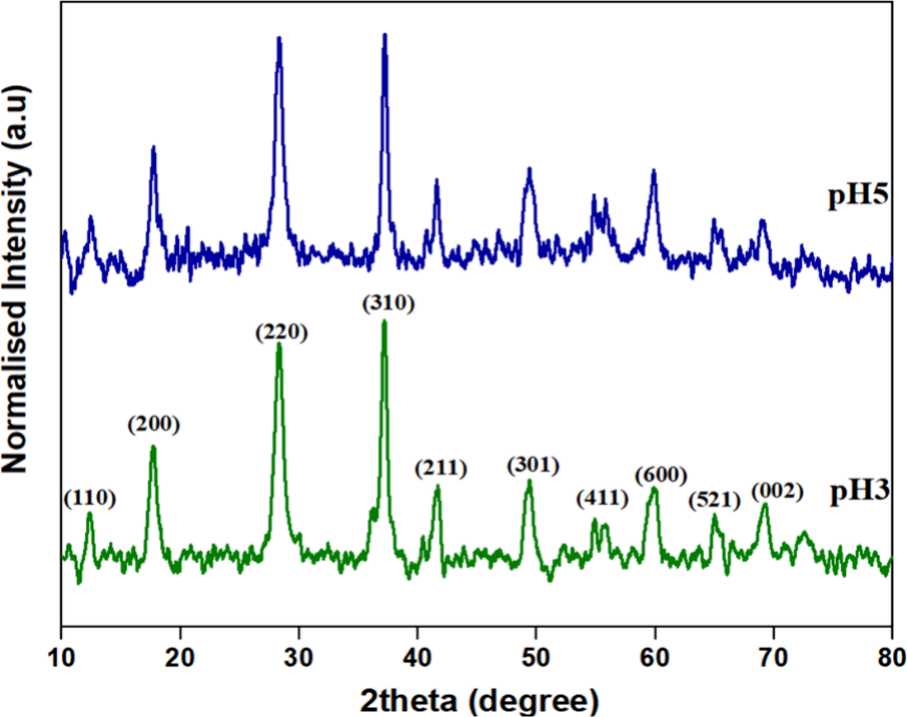
XRD pattern of pH3 and pH5.

**Table 1:** Structural Parameters of Synthesized Samples From XRD Analysis

sample name	*a* = *b* (Å)	*c* (Å)	phase	volume (Å^3^)	*D* (nm)
pH3	9.98	2.9	tetragonal	285.75	12.16
pH5	9.96	2.86		284.83	10.57

Figure 2 shows the surface morphology of the synthesized catalyst. Both catalysts have nanowire-like morphology. These results are consistent with the characteristic morphology of K-OMS-2. According to the literature, the precipitation of K-OMS-2 involves two primary processes. Initially, a layered or disordered precursor of manganese oxide is formed. Subsequently, this precursor undergoes a dissolution–recrystallization process to transform into fibrous K-OMS-2. Primary MnO_2_ crystallites are formed during the dissolution of the precursor in an acidic solution. Various experimental factors, such as temperature, pH level, and reactant concentration, significantly influence the crystal structure and morphology of the final product. In this study, the morphology of the two samples was found to be identical.^17–19^

**Figure 2:**
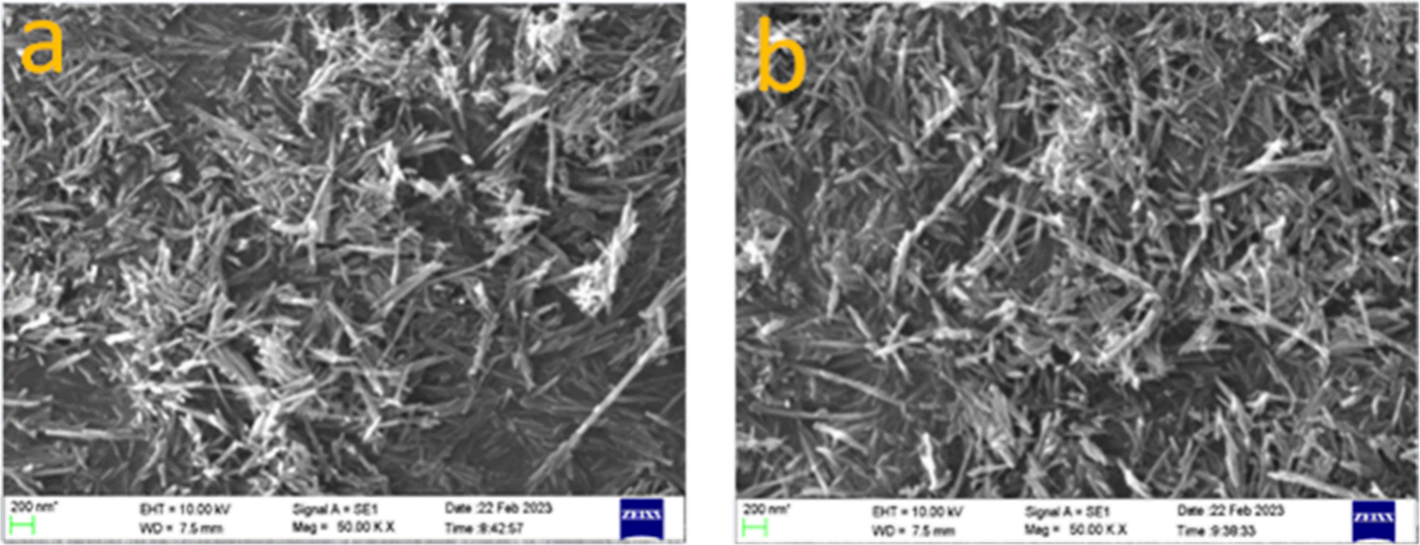
SEM images of (a) pH3 and (b) pH5.

### 3.2 FTIR Analysis

Figure 3 presents the FTIR spectra of the synthesized samples. The distinctive peaks characteristic of K-OMS-2 are observed around 3730, 1633, 1517, 771, 600, 516, and 452 cm^–1^, originating from the Mn–O vibrations of the MnO_6_ octahedra within the framework. The band at ∼3730 cm^–1^ is attributed to the stretching vibrations of structural – OH groups, while the bands at ∼1633 and 1500 cm^–1^ correspond to the bending vibrations of −OH groups from adsorbed water molecules within the tunnel structure of K-OMS-2. The prominent band observed at ∼771 cm^–1^ is assigned to the Mn–O–Mn stretching vibration of MnO_6_ octahedra, confirming the formation of the cryptomelane phase. Additional bands at ∼600, 516, and 452 cm^–1^ are associated with Mn–O lattice vibrations.^20–22^

**Figure 3:**
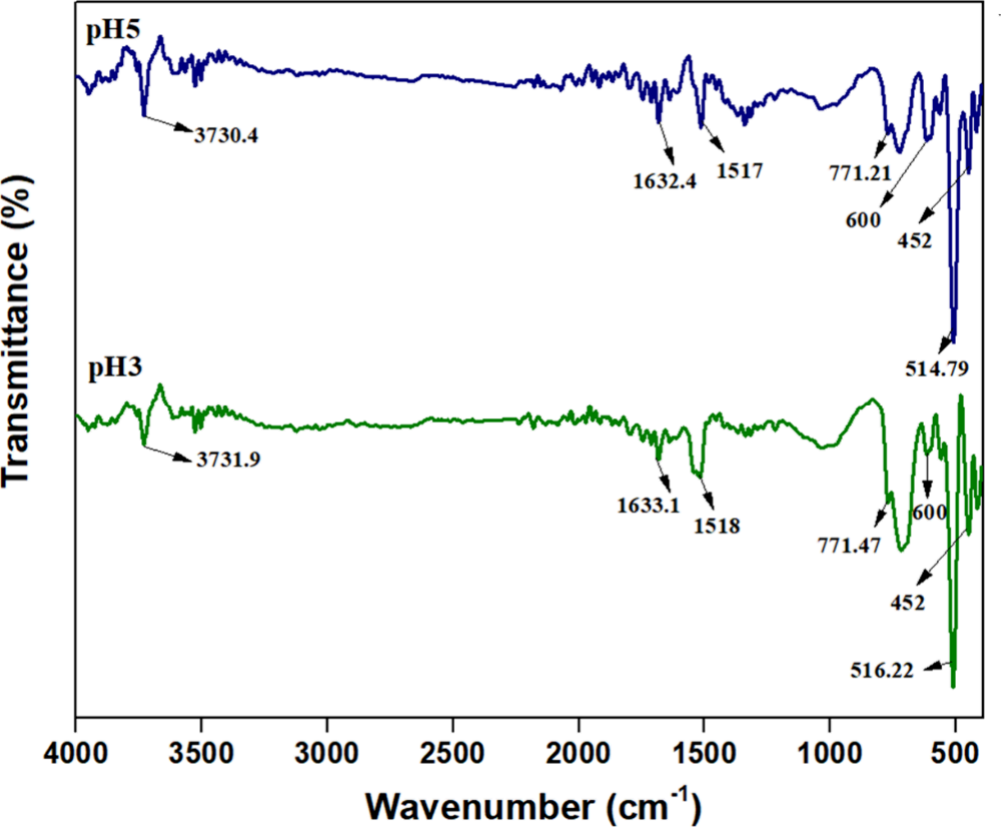
FTIR spectra of pH3 and pH5.

### 3.3 Raman Analysis

Figure 4 depicts the Raman spectra obtained from the synthesized samples. The observed characteristic bands at 183, 223, 281, 332, 391, 578, and 637 cm^–1^ are attributed to the Mn–O lattice vibrations within the tunnel structure of K-OMS-2. Specifically, the 183 and 223 cm^–1^ bands correspond to the translational motion of MnO_6_ octahedra. The 332, 391, and 738 cm^–1^ bands signify bending vibrations of the Mn–O bonds. The presence of the 578 cm^–1^ band indicates displacement of the O_2_ atoms relative to the manganese atoms along the octahedral chain. Finally, the 637 cm^–1^ band arises from the antisymmetric stretching of Mn–O vibrations. The main bands 578 and 637 cm^–1^ of the pH3 sample are symmetrical and sharp, indicating a higher order of crystallinity and fine particle size, which concords with XRD results.^23–25^

**Figure 4:**
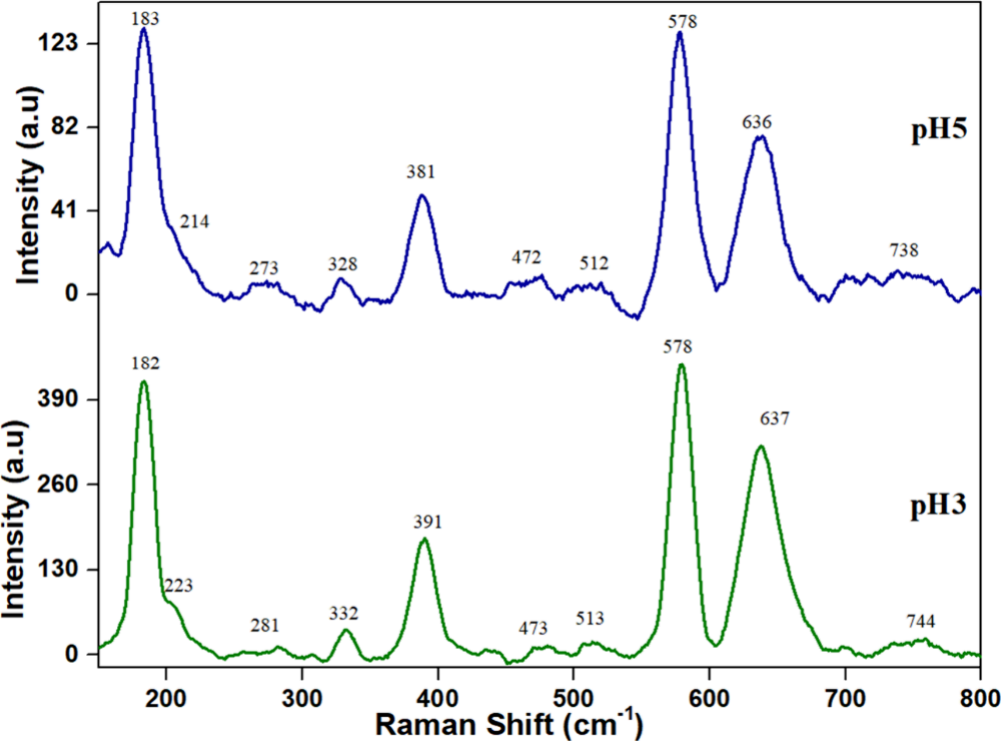
Raman spectra of pH3 and pH5.

### 3.4 XPS Analysis

The oxidation states and surface composition of the samples were analyzed by using XPS. As shown in Figure 5a,b, two distinct peaks at binding energies of 294 and 291 eV correspond to K2P_1/2_ and K2P_3/2,_ respectively, which are consistent with reported literature.^26^ For manganese, the Mn 2p spectra (Figure 5e,f) exhibit peaks at approximately 653.4 and 641.9 eV, assigned to Mn2*P*_1/2_ and Mn2P_3/2_, respectively, in agreement with literature values.^27^ The peaks around 655 and 643 eV are attributed to Mn^3+^, while those around 653 and 641 eV are attributed to Mn^4+^, confirming the presence of mixed valence states in K-OMS-2. The Mn^3+^/Mn^4+^ ratio, calculated from peak area analysis and provided in Table 2, is a crucial indicator of redox behavior. As reported by Wang,^28^ a higher surface concentration of Mn^3+^ induces oxygen vacancies, which can enhance the migration of active oxygen species and promote oxidation reactions such as soot combustion. Figure 5c,d shows the O 1s spectra, which were deconvoluted into three primary components. Peaks at ∼529, ∼530, and ∼531 eV are attributed to lattice oxygen (O_latt_) and chemisorbed (surface-adsorbed) oxygen species (O_ads_). The O_ads_/O_latt_ ratio, calculated from the peak area, is also listed in Table 2. The pH5 sample exhibits a higher proportion of surface-adsorbed oxygen, likely due to the greater presence of surface oxygen vacancies. These oxygen species play a critical role in soot oxidation by enhancing the redox cycling and lowering the reaction temperature.

**Figure 5:**
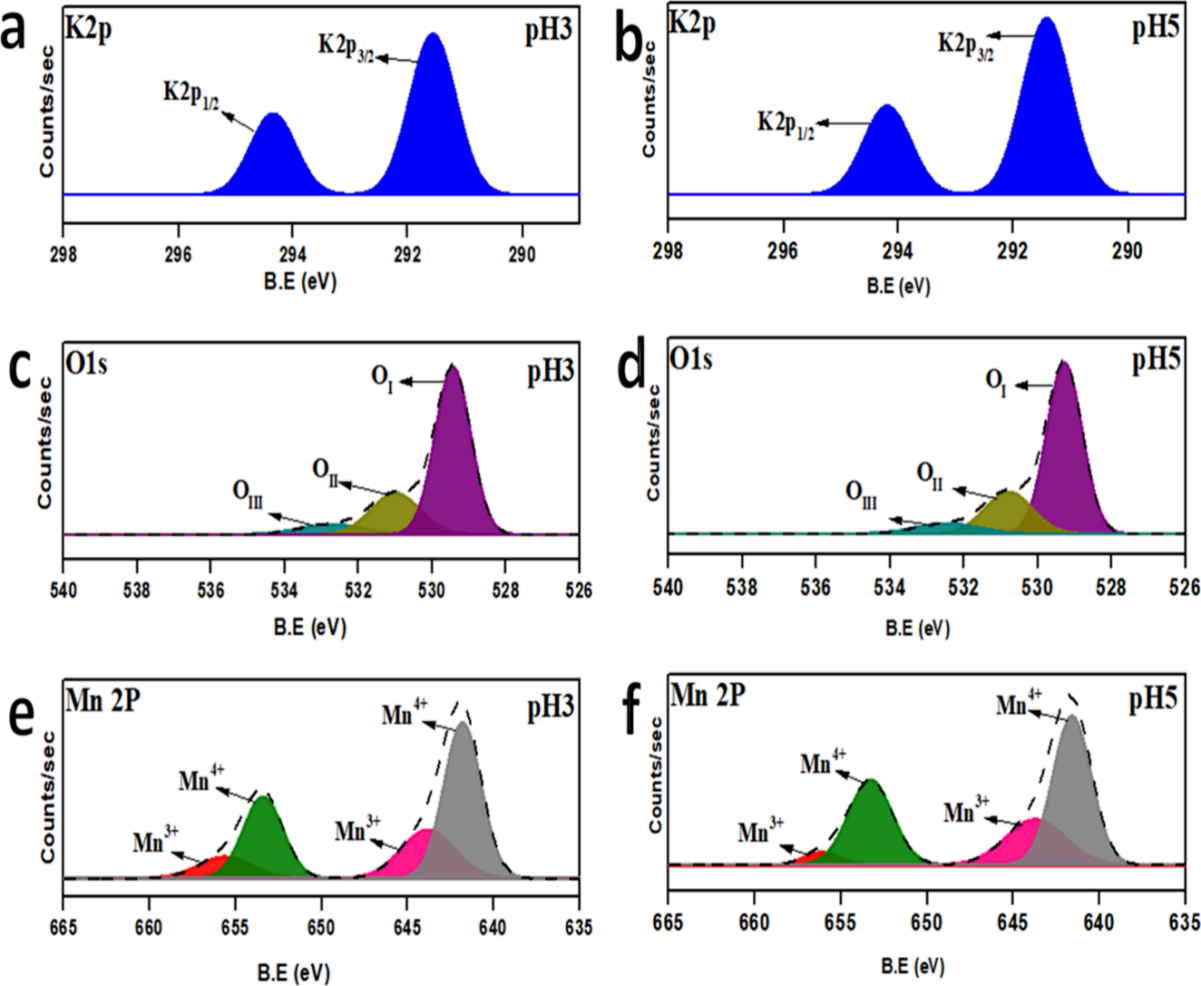
XPS spectra of pH3 and pH5 (a, b) K 2p (c, d) O 1s, and (e, f) Mn 2p.

**Table 2:** Binding Energies of O 1s

sample	element	B.E. (eV)	area	O_ads_/O_latt_ ^a^	Mn^3+^/Mn^4+^
pH3	O_III_	531.4	67926.5	0.40	0.38
	O_II_	530.8	60483.8		
	O_I_	529.4	188740.3		
pH5	O_III_	531.2	81446.53	0.86	0.41
	O_II_	530.7	75813.17		
	O_I_	529.3	23720		

a =O_ads_/O_latt_ (active species) = (O_III_ + O_II_)/(O_I_ + O_II_ + O_III_).

### 3.5 NH_3_-TPD Analysis

The surface acid properties were determined by using the NH_3_-TPD technique. Figure 6 displays the NH_3_-TPD profile of the synthesized catalysts. The pH3 catalyst exhibits three peaks, whereas pH5 exhibits a single peak. The peak centered between 100 and 300 °C is attributed to weak acidic sites, and the peak above 300 °C is attributed to moderate acidic sites. The peak around 600 °C is attributed to strong acidic sites. The role of weak acidic sites in soot oxidation remains less clear. However, in some catalysts, an increase in weak acidic sites is observed alongside a decrease in strong acidic sites, suggesting a possible trade-off in catalytic performance.^29^ Since strong acidic sites are often linked to the activation of reactant molecules, their reduction may influence the overall oxidation efficiency. Therefore, the pH5 catalyst, with fewer strong acidic sites, may exhibit altered catalytic behavior compared to pH3.

**Figure 6:**
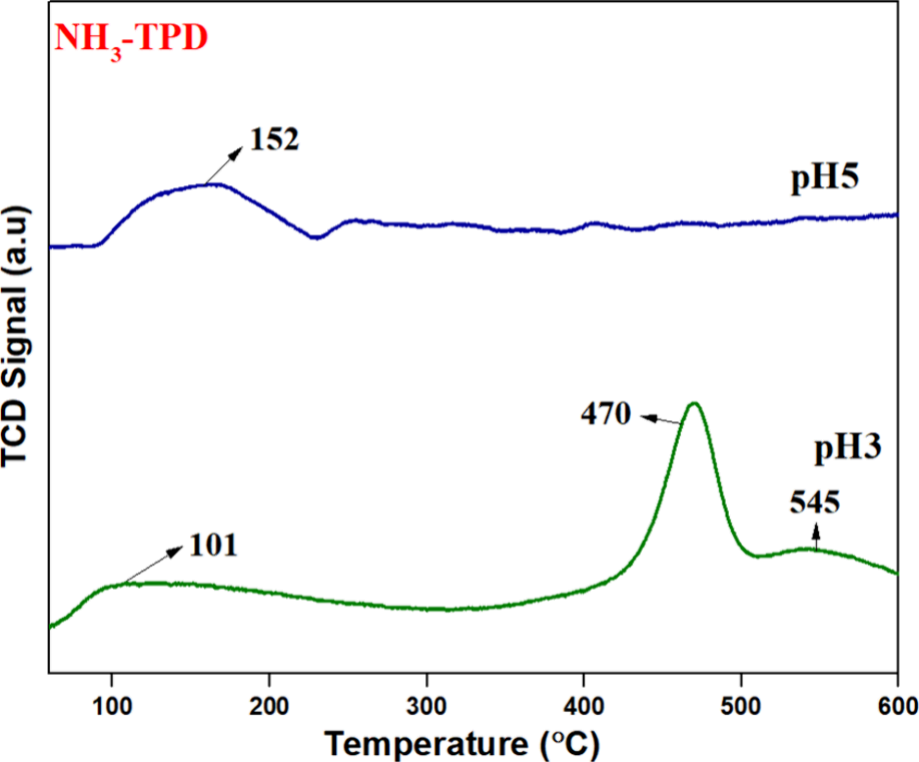
NH_3_-TPD of pH3 and pH5.

### 3.6 CO_2_-TPD Analysis

Figure 7 displays the CO_2_-TPD profile of the synthesized samples. The surface basicity of the catalyst can be analyzed by monitoring different temperatures of CO_2_ desorption. Figure 7 shows that the pH3 catalyst exhibits a single desorption peak, whereas the pH5 catalyst displays four distinct peaks. The peaks centered below 200 °C are attributed to weak basic sites, and peaks above 200 °C are attributed to moderate basic sites. However, no fixed temperature range strictly defines the strength of the basic sites. The classification is based on the catalyst composition, surface properties, and experimental conditions. Shang^30^ reported that weak basic sites contribute minimally to the soot oxidation process compared to moderate basic sites. This is because the formation of active oxygen species and surface oxygen vacancies, both essential for efficient soot oxidation, is not significantly promoted by weak basic sites. Due to the presence of more moderate basic sites, the pH5 catalyst is expected to exhibit superior catalytic activity in soot oxidation.

**Figure 7:**
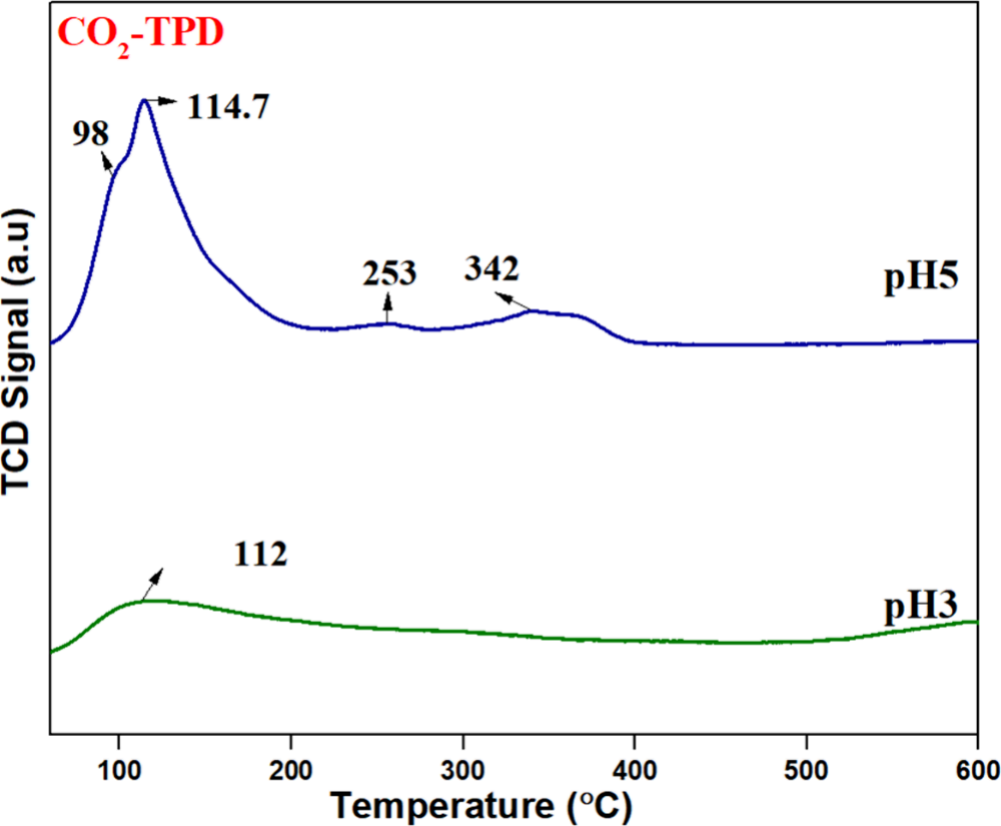
CO_2_-TPD of pH3 and pH5.

### 3.7 H_2_-TPR Analysis

The redox properties of the catalysts were measured through H_2_-TPR; the obtained profile is displayed in Figure 8. The reduction peak observed between 200 and 400 °C is due to the reduction of Mn^4+^ and Mn^3+^ ions to Mn^3+^ and Mn^4+^ ions.^31,32^ The reduction that occurred below 400 °C suggests the high oxygen mobility and surface-active oxygen species’ catalytic ability to oxidize soot at lower temperatures. H_2_ consumption analysis revealed that pH5 exhibited a significantly higher H_2_ uptake (19.313 mmol/g) compared to pH3 (6.157 mmol/g). This suggests that the pH5 sample contains a greater quantity of reducible oxygen species, as indicated by its Mn^3+^/Mn^4+^ redox activity, which may enhance its oxygen storage capacity and redox cycling ability. The higher H_2_ consumption at pH 5 also suggests the presence of more active oxygen species, which can play a crucial role in catalytic soot oxidation. Although pH3 exhibited a lower reduction temperature (112 °C), often linked to improved catalytic efficiency at lower operating temperatures, the higher H_2_ consumption in PH5 suggests that it possesses a larger reservoir of oxygen species, potentially sustaining oxidation reactions for a longer duration.

**Figure 8:**
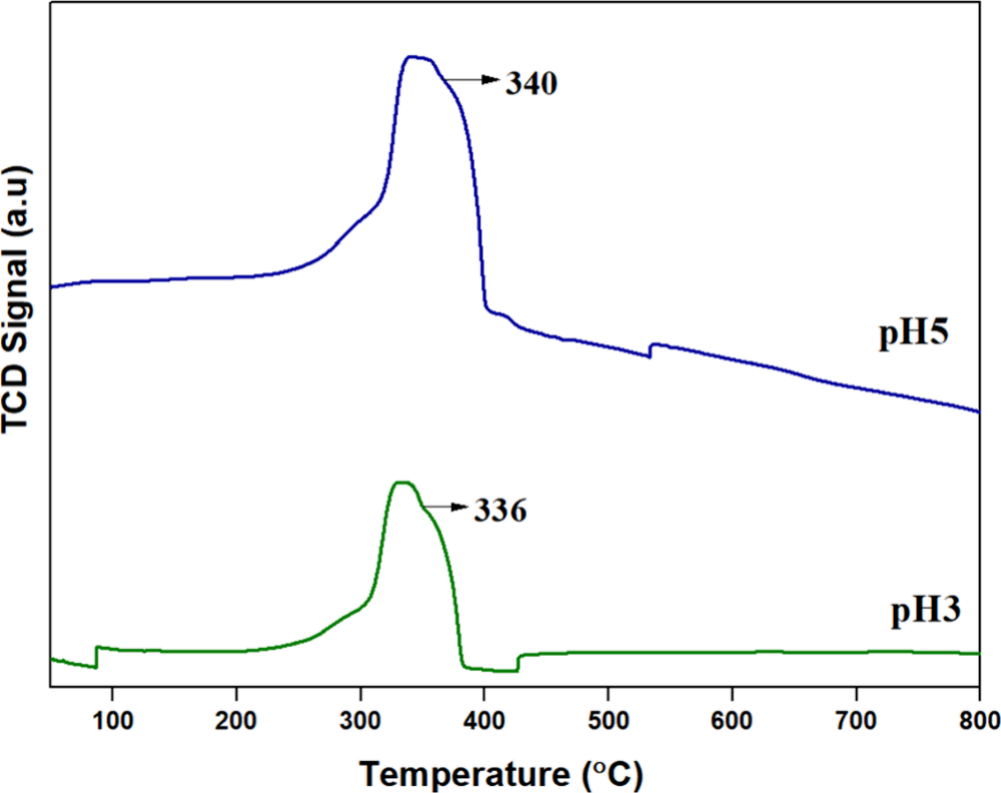
H_2_-TPR profile of pH3 and pH5.

### 3.8 Soot TPR

Figure 9 presents the soot-TPR analysis of the synthesized samples. This analysis aids in examining the role of various oxygen species in the soot oxidation process. Two types of oxygen species are evolved during this process. (i) The surface-adsorbed oxygen species evolved at a lower temperature range of 200–500 °C. These oxygen species are loosely bound to the surface of the catalysts and, thus, are readily released at lower temperatures. (ii) Lattice oxygen is released at above 500 °C; these species are not easily released from the catalyst.^33^ The lattice oxygen species must be activated, and reactive oxygen species are formed along with oxygen vacancies. These vacancies are subsequently replenished with oxygen, facilitating the restoration of lattice oxygen and the reoxidation of the catalyst. As previously discussed in the XPS-O 1s analysis, the pH5 sample showed a higher amount of active oxygen species. Similarly, the soot-TPR analysis also yielded similar results.

**Figure 9:**
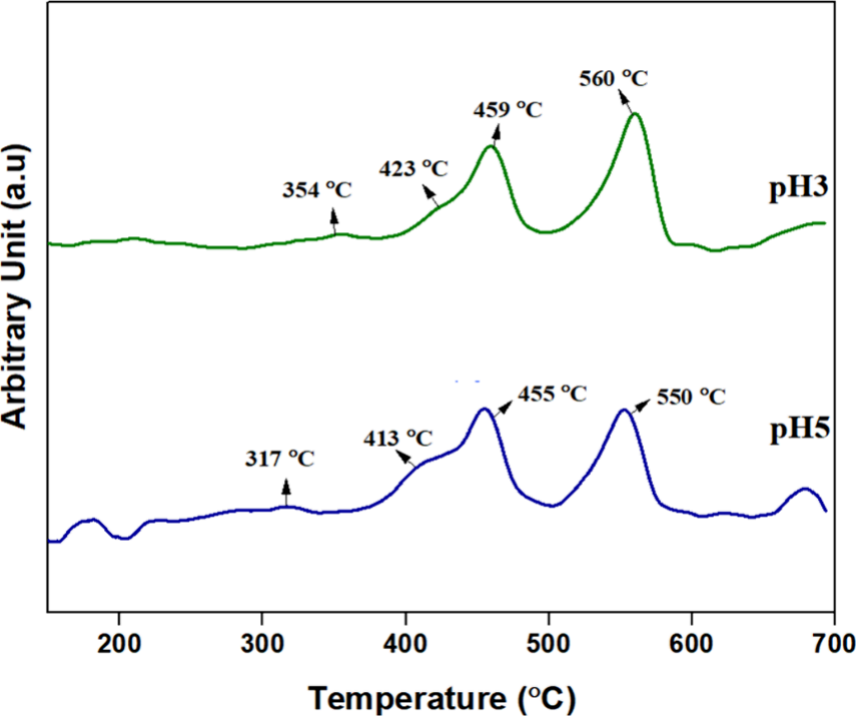
Soot TPR of pH3 and pH5.

### 3.9 Catalytic Activity–Soot Oxidation

To evaluate the catalytic efficiency of the synthesized K-OMS-2 samples, soot oxidation experiments were conducted using a 1:10 soot-to-catalyst ratio under a 5% O_2_ (balance N_2_) atmosphere to ensure close contact. The catalytic performance is presented in Figure 10. As expected, soot oxidation in the presence of the catalysts occurred at significantly lower temperatures compared with uncatalyzed combustion. Key performance indicators, *T*_50%_ and *T*_90%_, were used to compare the catalysts. The pH5 sample exhibited a *T*_50%_ of 368 °C, representing a substantial reduction of approximately 224 °C compared to the oxidation temperature of bare soot.

**Figure 10:**
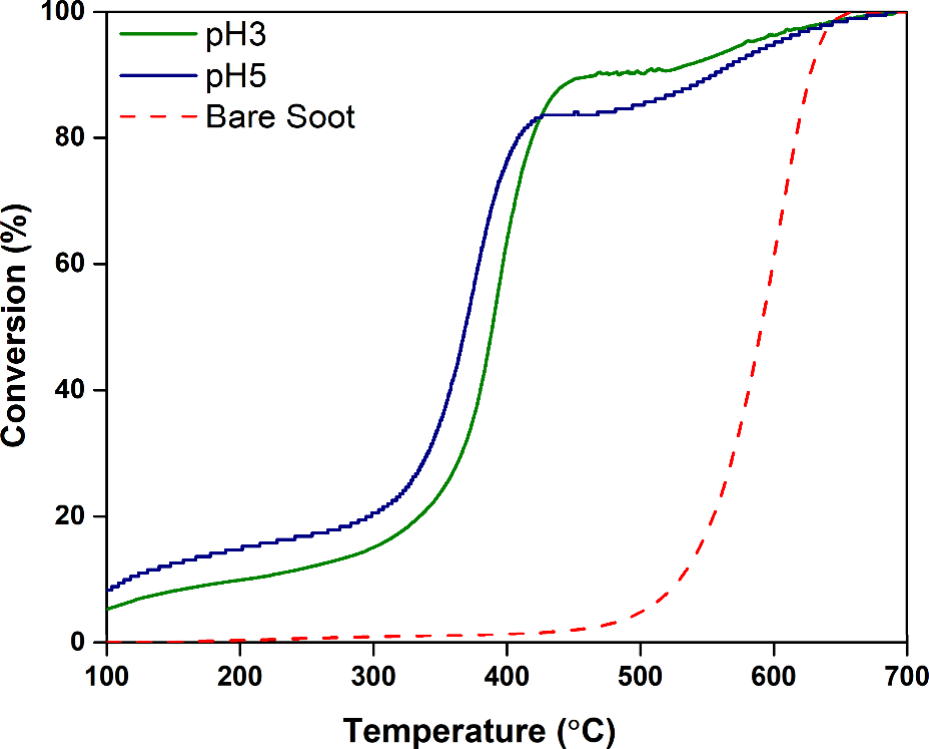
Soot oxidation of pH3 and pH5.

Effective low-temperature soot oxidation is generally associated with lower soot-TPR peak temperatures, enhanced redox properties, and a higher concentration of surface-adsorbed oxygen species. Soot-TPR analysis showed that both pH3 and pH5 samples displayed relatively low TPR peak temperatures; however, the pH5 sample demonstrated a higher concentration of chemisorbed oxygen, contributing to its superior catalytic activity and lower *T*_50%_.

Surface-adsorbed oxygen species are known to participate readily in oxidation reactions, as they are more labile and can interact with soot particles at lower temperatures. XPS analysis further confirmed the higher concentration of reactive oxygen species in the pH5 sample. Additionally, the improved Mn^3+^/Mn^4+^ redox balance in this sample promotes oxygen activation and regeneration, which are essential for sustained catalytic activity.

The role of oxygen species in soot oxidation can be explained as follows:

a. O_2_ (gas) → O_2_^–^/O^–^ (adsorbed on basic sites);
b. O^–^ (adsorbed) + Mn^3+^ (solid) → Mn^4+^–O^–^ (solid,active lattice oxygen);
c. C (solid, soot) + O^–^ (solid)→ CO/CO_2_ (gas) + □_O_ (solid);
d. Mn^3+^/Mn^4+^ redox transitions facilitate oxygen mobility (solid);
e. □_O_ (solid) + 1/2O_2_ (gas) → O_latt_ (solid).

In accordance with the mechanistic framework described in Section 3.8 of ref 34, soot oxidation over K-OMS-2 can be explained through the synergistic roles of surface-adsorbed oxygen, lattice oxygen, and oxygen vacancies. Molecular oxygen is initially adsorbed and activated at basic sites on the catalyst surface, generating reactive superoxide (O_2_^–^) and/or peroxide (O^–^) species. These species readily oxidize soot particles to aqueous CO and CO_2_. Simultaneously, lattice oxygen contributes to the oxidation process via the Mn^3+^/Mn^4+^ redox cycle, which facilitates oxygen migration to the surface while creating oxygen vacancies. These vacancies act as diffusion channels for oxygen transport and are subsequently replenished by gaseous O_2_, thereby restoring lattice oxygen. Thus, the process follows a Mars–van Krevelen mechanism in which the continuous interaction between surface oxygen species, lattice oxygen, and the Mn^3+^/Mn^4+^ redox couple sustains catalytic activity and ensures efficient oxygen regeneration.

The surface acidity and basicity of the catalysts also play important roles in the oxidation process. Acidic sites, as identified by NH_3_-TPD, can enhance the activation and adsorption of oxygen species on the catalyst surface. These activated oxygen species can then participate in oxidation reactions with soot particles. Additionally, the acidic environment can facilitate better interaction between the catalyst and the carbonaceous surface of soot, promoting more effective contact during oxidation.^35^

On the other hand, basic sites, revealed by CO_2_-TPD analysis, contribute to the formation of carbonate-like surface intermediates (C–O–M species, where M is a metal ion). These intermediates are reactive toward oxygen and promote the oxidative breakdown of soot. Furthermore, basic sites can enhance the mobility and availability of surface oxygen species, which is critical for achieving efficient soot oxidation at lower temperatures.^36^

The presence of Mn^3+^ ions in MnO_6_ octahedra introduces structural distortions that enhance redox behavior. Similar observations have been reported in the literature^37^ for catalysts such as LiMn_2_O_4_, where slight deviations from equilibrium positions during Mn^3+^ ⇌ Mn^4+^ transitions are associated with enhanced catalytic performance. Such distortions likely contribute to the improved activity of the pH5 K-OMS-2 catalyst.

A possible reaction mechanism is proposed (Figure 11):

a. Adsorption of Soot
  C_soot_ (solid) + acidic site → C_adsorbed_ (adsorbed)
b. Activation of Oxygen
  O_2_ (gas) + basic site → O^–^ (adsorbed) + basic site
  These activated O^–^ species can migrate to soot–catalyst interfaces.
c. Formation of Surface Intermediates (via Basic Sites)
  C_adsorbed_ (adsorbed) + O^–^ (adsorbed) → C–O–M_intermediate_ (adsorbed)
  These intermediates can be further oxidized to gaseous products.
d. Oxidation of Soot via Lattice Oxygen (MvK mechanism)
  C_adsorbed_ (adsorbed) + O_latt_ (solid) → CO/CO_2_ (gas) + □_O_ (solid)
  (where □_O_ is oxygen vacancy)
e. Mn^3+^/Mn^4+^ Redox Transition
  Mn^4+^ (solid) + e^–^ → Mn^3+^ (solid)
f. Catalyst Reoxidation
  □_O_ (solid) + 0.5 O_2_ (gas) → O_latt_ (solid); Mn^3+^ (solid) → Mn^4+^ (solid)

**Figure 11:**
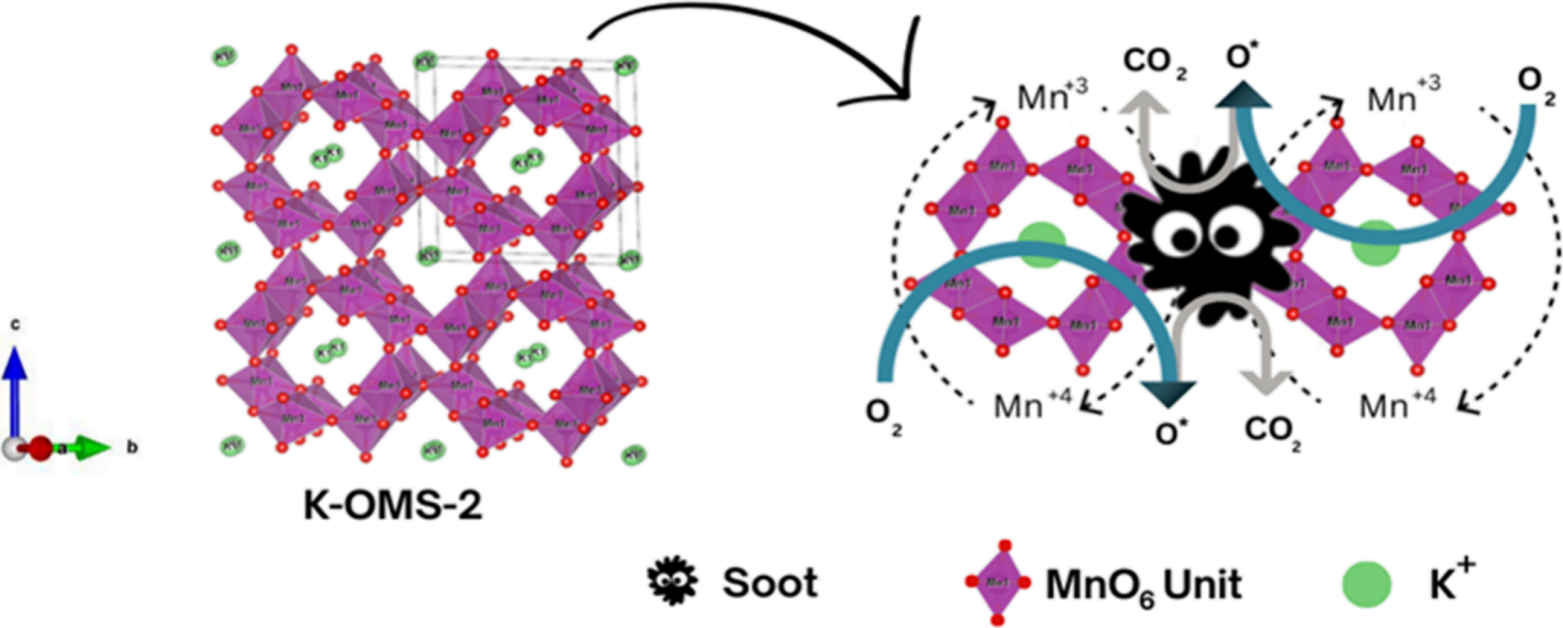
Possible reaction mechanism.

The soot oxidation mechanism over K-OMS-2, involving both acid–base surface interactions and the Mars–van Krevelen pathway,^38^ is influenced by the pH during synthesis. Although the pH3 and pH5 samples exhibit a similar nanowire-like morphology, the pH5 sample demonstrates superior catalytic activity due to enhanced surface properties. Specifically, the pH5 sample shows a higher density of acidic and basic surface sites, as confirmed by NH_3_-TPD and CO_2_-TPD analyses. Acidic sites facilitate soot adsorption, while basic sites enhance oxygen activation and promote the formation of reactive C–O–M surface intermediates. Furthermore, XPS analysis revealed a higher Mn^3+^/Mn^4+^ ratio and greater surface-adsorbed oxygen species in the pH5 sample, which support more efficient redox cycling and lattice oxygen participation in soot oxidation. In contrast, the pH3 sample has lower concentrations of these active features, resulting in reduced oxidation efficiency. Therefore, the enhanced performance of the pH5 sample can be attributed to a more favorable balance of surface acidity/basicity and redox-active oxygen species, which together facilitate the multistep mechanism for soot oxidation.

#### Correlation of the Proposed Mechanism with Kinetic Models

a. Diffusion-controlled components (D-models):
  Migration/transport of reactive oxygen (O_2_^–^/O^–^) to the soot–catalyst interface and lattice-oxygen replenishment via vacancy diffusion correspond to the D1–D3 regime for the pH3 catalyst, indicating short-to-intermediate diffusion lengths through the near-surface region. For pH5, the agreement extends to D4, consistent with longer-range diffusion enabled by its higher concentration of surface-adsorbed oxygen and enhanced oxygen mobility (as supported by NH_3_-TPD/CO_2_-TPD and XPS).
b. Surface-reaction component (R2):
  The oxidation of adsorbed carbon by activated/surface or lattice oxygen at the interface aligns with the R2 (second-order) rate form, reflecting the bimolecular interaction between reactive oxygen species and surface carbon sites.

Collectively, pH3 (D1–D3 + R2) indicates a mechanism in which oxygen diffusion is required but limited to shorter paths, operating in tandem with a surface reaction step. In contrast, pH5 (D1–D4 + R2) exhibits stronger diffusion assistance, including longer-range transport, together with the same R2 surface reaction, explaining its superior activity.

### 3.10 Determination of Activation Energy

FWO is a model-free and isoconversional technique used to determine the activation energy without the need for the assumption of a reaction model. The obtained values are tabulated in Table 3, and the visual representation is presented in Figure 12. The pH5 catalyst had a lower activation energy of 130.07 kJ/mol. The activation energy of uncatalyzed soot oxidation ranges from 180 to 200 kJ/mol.^39,40^ Compared to the activation energy of ∼195 kJ/mol for uncatalyzed soot oxidation reported in the literature,^41^ the K-OMS-2 catalyst synthesized at pH5 significantly reduced the apparent activation energy to ∼130 kJ/mol. The activation energy influences the efficiency of the catalyst. Lower activation energy suggests that the catalyst can facilitate the soot oxidation reaction at a lower temperature.^42^ For instance, Dhakad^43^ reported that the activation energy of soot oxidation decreased from 163 to 140 kJ/mol when Co_3_O_4_/CeO_2_ catalyst was employed. The activation energies vary with the different types of catalysts and their composition. Jian et al.^44^ synthesized CeO_2_ with varying morphology and observed that the nano cube-shaped CeO_2_ exhibited the lowest activation energy and the highest performance toward soot oxidation.

**Table 3:** Kinetic Parameters of pH3 and pH5 Samples

	activation energy (kJ/mol)		avg. pre-exponential factor (min^–1^)			
sample	**FWO**	**CR**	**Am**	**CR**	Avrami integer	reaction model^a^
pH3	153.67	153.43	24.58	14.73	0.35	D1, D3, D2, R2, D5
pH5	130.07	129.62	20.57	14.08	0.32	D1, D3, D4, D2, R2

a Where, D1 = 1-D Diffusion, D2 = 2-D Diffusion, D3 = 3-D Diffusion, D4 = Ginstling–Brounshtein equation, D5 = Zhuravlev equation, R2 = second-order chemical equation.

**Figure 12:**
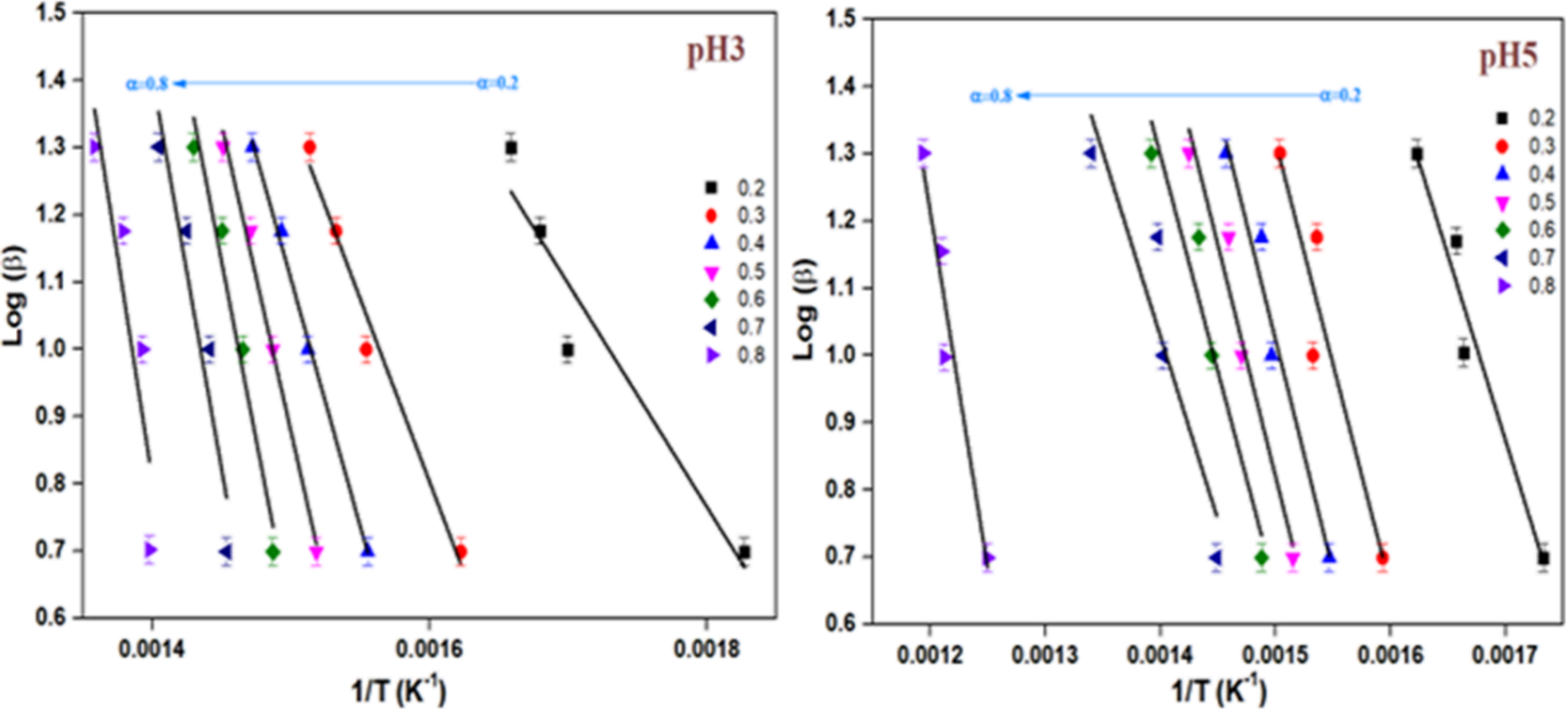
FWO plots of synthesized samples.

### 3.11 Determination of Pre-Exponential Factor

The estimation of the pre-exponential factor is a crucial parameter in the kinetics of soot oxidation, as it provides insight into the probability of collisions between soot and the catalyst. The Avrami–Erofeev (Am) model was employed to determine the pre-exponential factor and *m* noninteger using the Am expression. Ideally, soot oxidation follows the nucleation and nuclei growth model (*m* = 1.5–4). However, under actual conditions, it deviates from this model considering particle size, shape, etc. Therefore, a noninteger value of m is used to accurately describe the real soot oxidation process.^45^ Figure 13 displays the Am plots, and the obtained *m* value ranges from 0.32 to 0.35 for the samples at pH5 and pH3, respectively. Lou et al.^46^ reported an A value of 6.37 × 10^7^ min^–1^ for the Pt–Pd-based catalyst. Wagloehner and Kureti^47^ also reported an *A* value of 1.6 × 10^3^ m^3^/mol·s for the catalyst Fe_2_O_3_. Therefore, it can be said that the *A* values differ with different catalysts.

**Figure 13:**
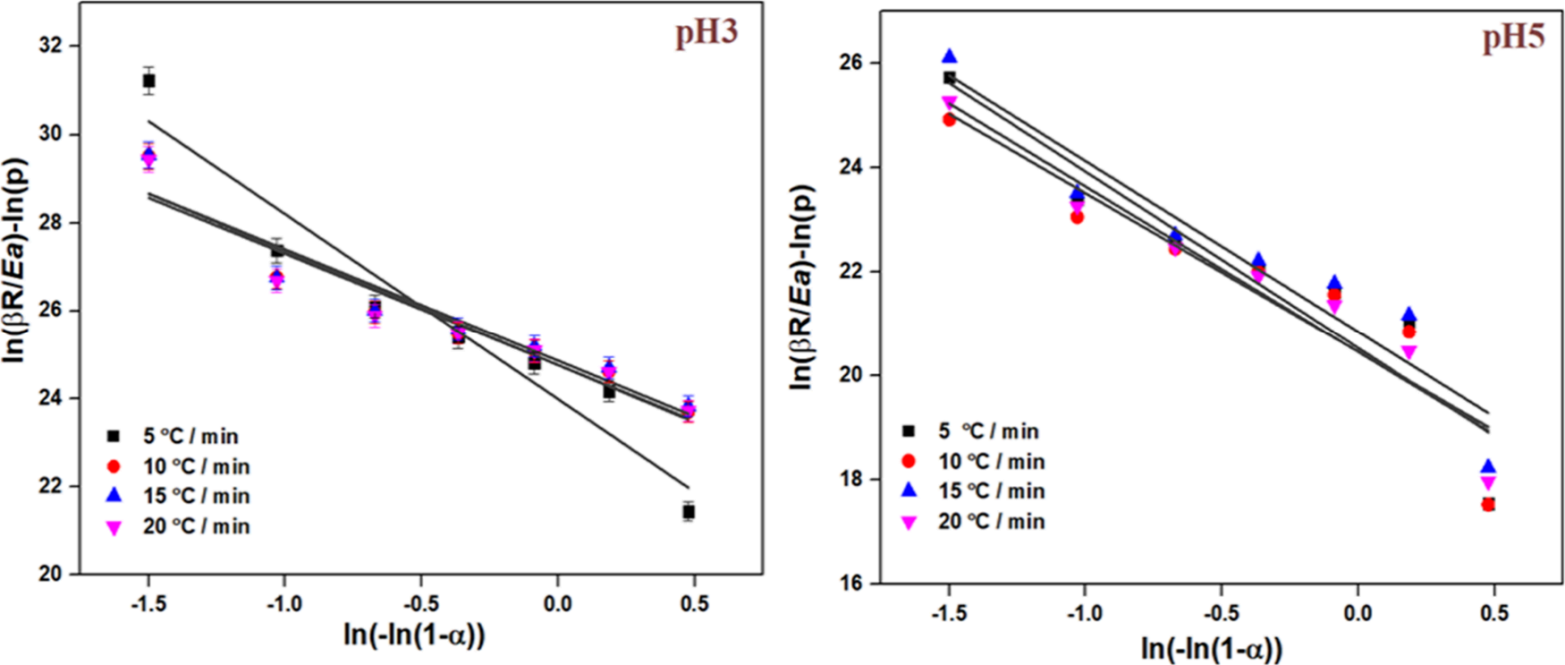
Am plots of synthesized samples.

### 3.12 CR Method

The CR plots were plotted to compare the obtained values of *E*_a_ and *A*. Figure 14 displays the CR plots for the synthesized catalysts. The obtained *E*_a_ and *A* values are listed in Table 3. The obtained *E*_a_ and *A* values were similar to those obtained via the FWO method.

**Figure 14:**
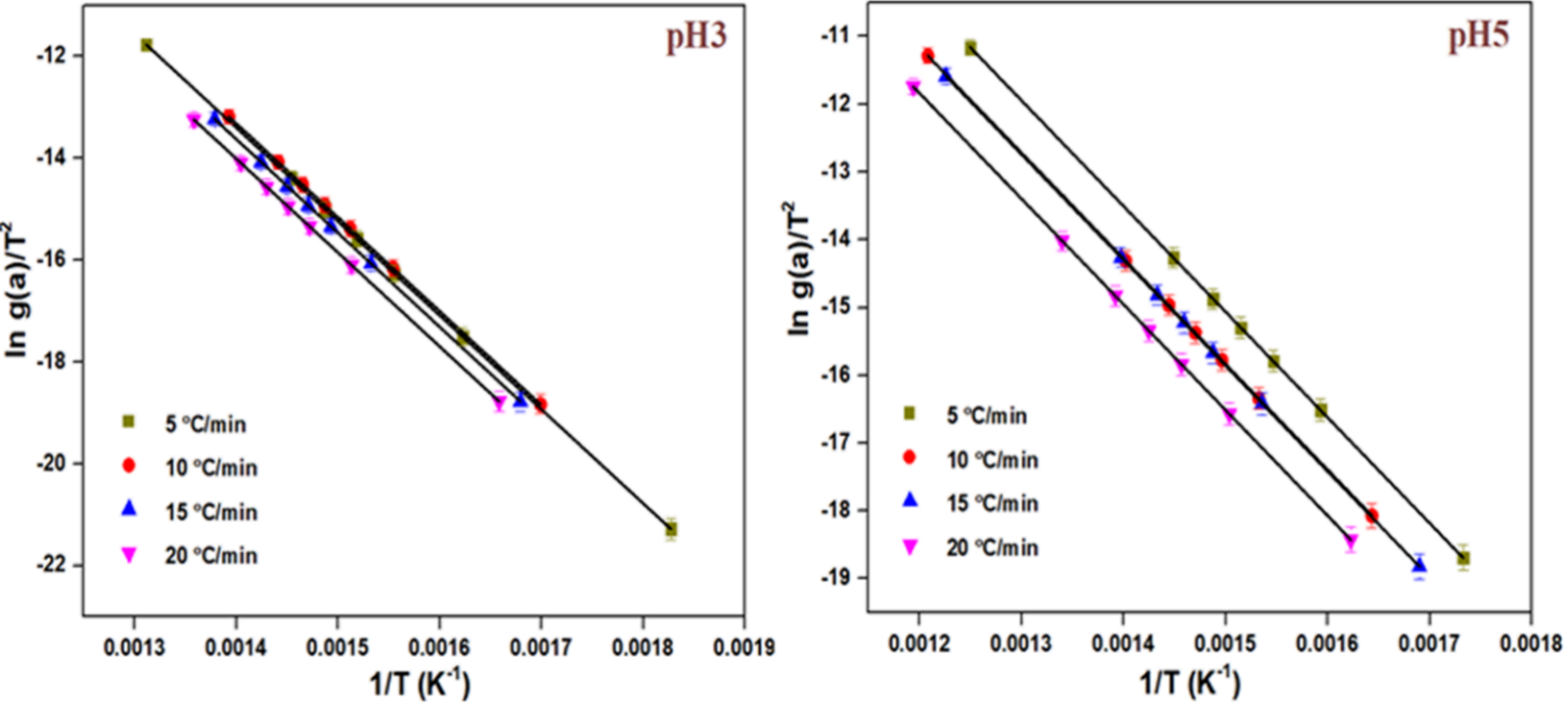
CR plots of synthesized samples.

### 3.13 Determination of Reaction Model

The appropriate physicochemical conversion models for soot oxidation were identified using the master plot method, which allows comparison of normalized experimental data with theoretical reference curves corresponding to different solid-state kinetic models. Master plots are theoretical functions *f*(α) or g­(α) that represent ideal kinetic behaviors (reaction-controlled, diffusion-controlled, geometrical contraction, etc.), and are generally independent of kinetic parameters such as activation energy. In this approach, the experimental conversion data were transformed into normalized master plots and compared against the theoretical plots for models D1–D4 (diffusion-controlled), R1–R3 (reaction-controlled), and A2–A3 (geometrical contraction). The best-fitting models were selected based on the close overlap of the experimental master plot with the theoretical curve.

Figure 15 depicts the master plots for the pH3 and pH5 samples corresponding to the *g*(α) functions as reported in López-Fonseca et al.^45^ As observed, sample pH3 follows the D1–3,5 and R2 model, and sample pH5 follows the D1–4 and R2 model. The R2 model closely relates to the nucleation and reaction interface progression.^48^ In the case of catalytic soot oxidation, soot oxidation starts when the active oxygen species are adsorbed onto the catalyst surface, acting as nucleation sites. Once oxidation starts, the reaction front moves from the outer surface of the soot particle toward its core. The oxidation process is controlled by the migration of oxygen species, the diffusion of reaction intermediates, and temperature conditions. Catalysts with high oxygen mobility and surface reactivity enhance nucleation and reaction progression, which decreases the oxidation temperature. The reaction interface moves efficiently when oxygen vacancies, redox-active sites, and spillover mechanisms facilitate oxygen transport. Whereas the D1–D5 model applies to diffusion-controlled processes.^49,50^ The D1–D5 solid-state reaction models are used to describe different diffusion-controlled mechanisms relevant to catalytic soot oxidation. In this process, soot oxidation is initiated by the reaction of surface oxygen species with soot (D1), followed by the lateral diffusion of oxygen across the surface (D2) and its subsequent penetration into the soot particle (D3). The oxidation process is often governed by a shrinking-core mechanism (D4), where the reaction front progresses inward as oxygen diffuses through the soot structure. In some cases, nonuniform diffusion (D5) is observed due to agglomeration or pore blockages, which restrict oxygen transport. The oxidation efficiency is enhanced by effective catalysts as they facilitate oxygen mobility, provide redox-active sites, and promote spillover mechanisms, enabling soot oxidation at lower temperatures. The pH3 sample followed the D1, D2, D3, D5, and R2 models, indicating that soot oxidation was primarily influenced by surface reaction, oxygen diffusion, and nonuniform transport pathways. The presence of the D5 model suggests diffusion limitations, likely due to structural factors affecting the oxygen mobility. In contrast, the pH5 sample followed the D1, D2, D3, D4, and R2 models, demonstrating a more uniform shrinking-core mechanism (D4) that facilitated sustained oxygen diffusion and reaction progression. Both samples exhibited the R2 model, signifying the role of nucleation and the reaction interface progression in soot oxidation. Overall, the pH5 catalyst showed a more efficient oxidation pathway with enhanced oxygen transport, suggesting its superior catalytic performance compared to pH3. The results are consistent with the XPS analysis, which showed that the pH5 sample contained the largest amount of active oxygen species. This suggests that pH5 is a more efficient catalyst for soot oxidation compared to pH3.

**Figure 15:**
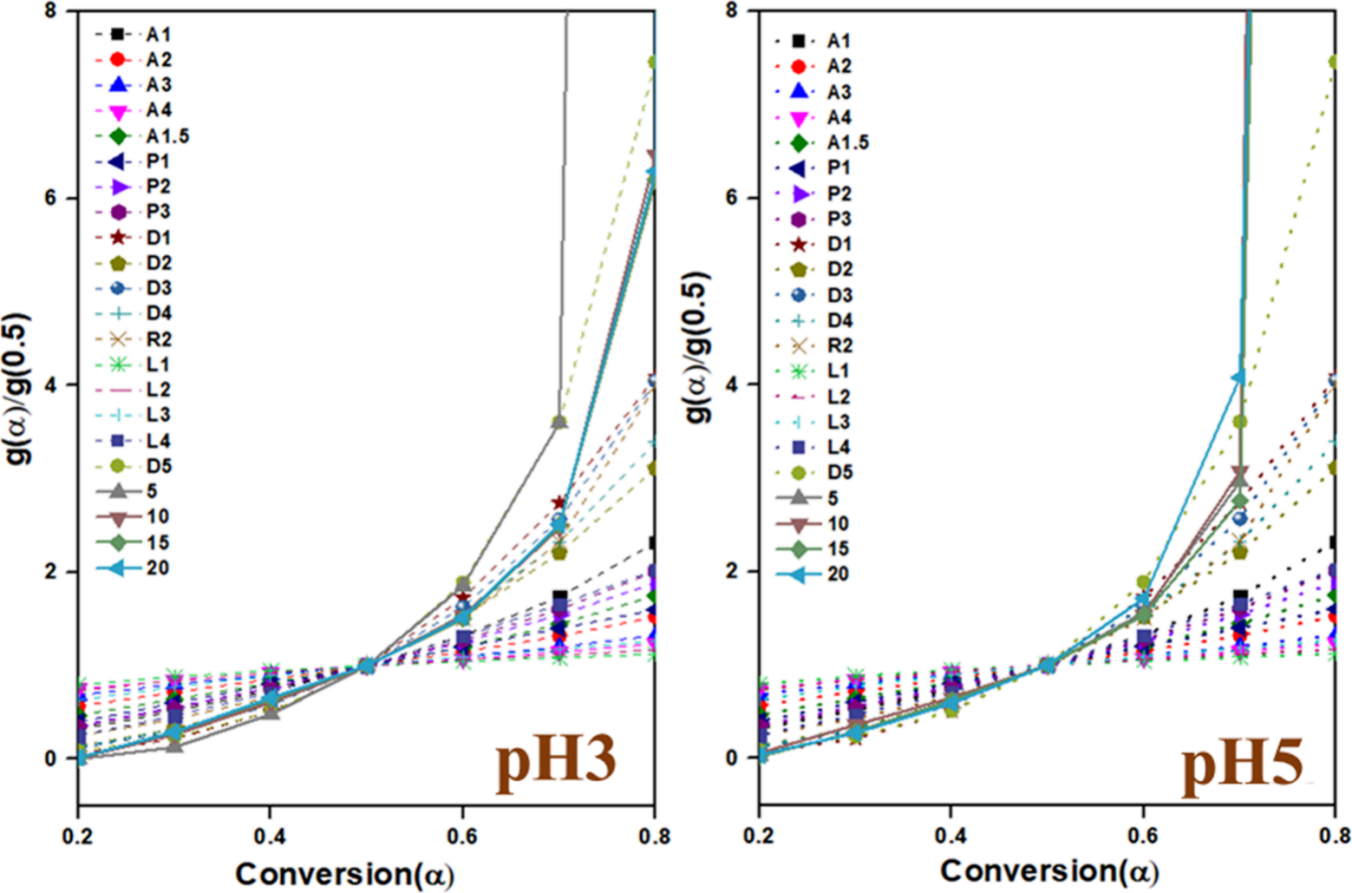
Master plots of synthesized samples.

### 3.14 Determination of Rate of Reaction and Kinetic Activity

The rate of reaction is determined by plotting dα/d*T* vs temperature, as shown in Figure 16b. The pH5 sample achieved the highest rate at high temperatures compared to that of the pH3 sample. It can also be said that the rate increases with an increase in the α value. The kinetic activity was determined by plotting ln­(*k*) vs 1/*T* as displayed in Figure 16a. As witnessed, the pH5 sample achieved the highest activity compared to the pH3 sample.

**Figure 16:**
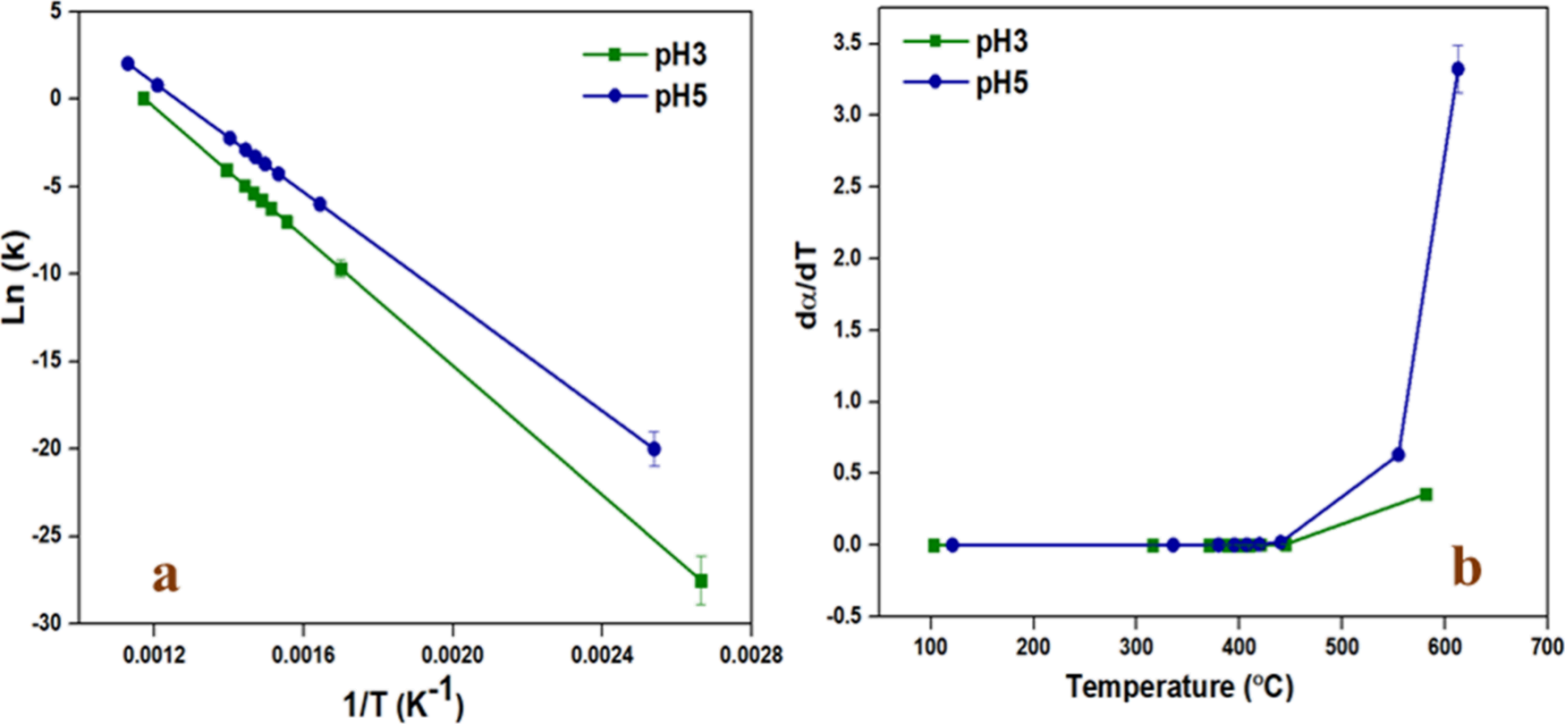
(a) Arrhenius plot at a heating rate of 10 °C/min; (b) rate vs temperature at a heating rate of 10 °C/min.

## 4 Conclusions

pH3 and pH5 samples were successfully synthesized via the hydrothermal method. The pH of the respective samples was adjusted accordingly. Both samples exhibited a tetragonal phase, with the pH5 sample exhibiting the smallest crystallite size of 10.57 nm. The SEM analysis revealed similar morphology, suggesting that the pH has not affected the sample’s morphology. The XPS analysis revealed that the pH5 sample had the highest amount of Mn^3+^ ions and active oxygen species. The highest amount of low-valence Mn ions is vital in improving oxygen vacancies and surface-adsorbed oxygen species. The acidic and basic sites of the pH3 and pH5 samples were evaluated using NH_3_-TPD and CO_2_-TPD experiments. The H_2_-TPR and soot-TPR analysis revealed the reducibility capacity of the synthesized samples. pH5 exhibited superior catalytic performance with a *T*_50%_ value of 368 °C. The activation energy of catalytic soot oxidation was much lower than that of bare soot oxidation. Interestingly, pH3 showed a high pre-exponential factor, suggesting the probability of soot and catalyst collisions is high for the pH3 sample. However, the master plots suggest that the pH5 sample showed efficient catalysts in terms of active oxygen mobility. The rate of reaction and kinetic activity were high for the pH5 sample.

## Data Availability

All data supporting the findings of this study are available within the article.
